# Harms of prescription opioid use in the United States

**DOI:** 10.1186/1747-597X-9-43

**Published:** 2014-10-27

**Authors:** Sameer Imtiaz, Kevin D Shield, Benedikt Fischer, Jürgen Rehm

**Affiliations:** Institute of Medical Science, University of Toronto, Toronto, Canada; Centre for Addiction and Mental Health, Toronto, Canada; Department of Psychiatry, University of Toronto, Toronto, Canada; Centre for Applied Research in Mental Health and Addiction, Simon Fraser University, Vancouver, Canada; Institute for Clinical Psychology and Psychotherapy, Technische Universität Dresden, Dresden, Germany; Dalla Lana School of Public Health, University of Toronto, Toronto, Canada

**Keywords:** Prescription opioid use, Non-medical use, Addiction treatment, Overdose mortality, Public health and pain treatment

## Abstract

**Background:**

Consumption levels of prescription opioids (POs) have increased substantially worldwide, particularly the United States. An emerging perspective implicates increasing consumption levels of POs as the primary system level driving factor behind the observed PO-related harms. As such, the present study aimed to assess the correlations between consumption levels of POs and PO-related harms, including non-medical prescription opioid use (NMPOU), PO-related morbidity and PO-related mortality.

**Findings:**

Pearson’s product-moment correlations were computed using published data from the United States (2001 – 2010). Consumption levels of POs were extracted from the technical reports published by the International Narcotics Control Board, while data for NMPOU was utilized from the National Survey on Drug Use and Health. Additionally, data for PO-related morbidity (substance abuse treatment admissions per 10,000 people) and PO-related mortality (PO overdose deaths per 100,000 people) were obtained from published studies. Consumption levels of POs were significantly correlated with prevalence of NMPOU in the past month (r =0.741, 95% CI =0.208–0.935), past year (r =0.638, 95% CI =0.014–0.904) and lifetime (r =0.753, 95% CI =0.235-0.938), as well as average number of days per person per year of NMPOU among the general population (r =0.900, 95% CI =0.625-0.976) and NMPOU users (r =0.720, 95% CI =0.165–0.929). Similar results were also obtained for PO-related morbidity and PO-related mortality measures.

**Conclusion:**

These findings suggest that reducing consumption levels of POs at the population level may be an effective strategy to limit PO-related harms.

## Background

Pain has been long regarded as a stepchild area of medicine until recently. Prescription opioids (POs) are mainly used as analgesics in the treatment of mild to severe pain, including cancer and chronic non-cancer pain. In the context of expanded pain care, their consumption levels have tripled globally since 1990; however, this expansion has occurred almost exclusively in high-income countries, particularly the United States, which has ranked highest in *per capita* consumption of POs (based on standardized doses) for the past decade [1].

Coinciding with this expansion in consumption levels of POs in the United States have been several PO-related harms, namely non-medical prescription opioid use (NMPOU), PO-related morbidity and PO-related mortality. For example, prevalence of NMPOU during the past year has risen to almost 5% over the past decade [2]. Similarly, substance abuse treatment admissions for POs have increased from 28,326 in 2000 to 157,171 in 2010 [3], whereas there has been a fourfold increase between 1999 and 2010 in the number of drug poisoning deaths involving POs [4]. Therefore, it is hardly surprising that PO-related deaths now surpass deaths related to heroin and cocaine use combined [5].

Given previous experiences with other psychoactive substances, one perspective on this area of research, supported by emerging evidence from the United States [6–8] and Canada [6, 9, 10], implicates increasing consumption levels of POs as the primary system-level driving factor behind the surging opioid epidemic. This short report aims to further this perspective by using published data for the United States from 2001 – 2010.

## Methods

### Data

#### Consumption levels of POs

Annual consumption levels of POs were derived from technical reports published by the International Narcotics Control Board (INCB), which detail the availability and use of narcotic substances in various countries based on information provided by international governments to the board [1]. Data within these reports were available in the form of defined daily doses for statistical purposes (S-DDD), which are technical units of measurements meant for statistical analysis, and which facilitate comparisons between different kinds of opioids based on their potency [1]. In short, calculation of this metric involves a series of succeeding divisions of the annual consumption of narcotic substances by 365, country population (millions) during a given year and defined daily dose [11]. Importantly, a given year is defined as the midpoint of its interval in the presentation of the data for this metric by the INCB [1]. For example, the estimate for 2010 represents the average of the estimates for the 2009 – 2011 interval [1].

#### PO-related harms: NMPOU, PO-related morbidity and PO-related mortality

Measures of NMPOU included annual prevalence of NMPOU during the past month, past year and lifetime, which were drawn from nationally representative surveys of the United States population: National Survey on Drug Use and Health Series [2]. These surveys define NMPOU as use without a prescription or use solely for the experiences or feelings induced by POs [2]. Additionally, two other measures targeting the extent of NMPOU occasions were also taken from these surveys, namely average number of days per year per person of NMPOU among the general population and among NMPOU users [2].

The PO-related morbidity and PO-related mortality measures were extracted from published studies [12, 13]. The PO-related morbidity measure was substance abuse treatment admissions for POs per 10,000 people [12], which was based on data from the Substance Abuse and Mental Health Services Administration’s Treatment Episode Data Set. Though this measure has been previously analyzed with S-DDD [6], it was included in the present study to provide a comprehensive overview of the harms of PO use in the United States. On the other hand, the PO-related mortality measure, PO overdose deaths per 100,000 people [12, 13], was computed using data from the National Vital Statistics System multiple cause-of-death file. Importantly, the 2009 estimate for this measure was based on an imputation procedure, as data was not available in published studies.

Table 1 presents the annual data from 2001 – 2010 for the consumption levels of POs, NMPOU, PO-related morbidity and PO-related mortality measures.

**Table 1:** **Consumption levels of prescription opioids, non-medical prescription opioid use, prescription opioid-related morbidity and prescription opioid-related mortality measures in the United States from 2001 – 2010**

Year	S-DDD	NMPOU prevalence (past month)	NMPOU prevalence (past year)	NMPOU prevalence (lifetime)	Average number of days per year per person of NMPOU (general population)	Average number of days per year per person of NMPOU (NMPOU users)	Substance abuse treatment admissions for POs per 10,000 people	PO overdose deaths per 100,000 people
**2001**	22,524	1.5%	3.7%	9.8%	1.61	43.93	1.31	1.92
**2002**	25,993	1.9%	4.7%	12.8%	1.91	40.55	1.58	2.57
**2003**	29,500	2.0%	5.0%	13.2%	1.92	38.33	1.81	2.90
**2004**	33,532	1.8%	4.7%	13.4%	1.85	39.46	2.08	3.33
**2005**	37,565	1.9%	4.8%	13.5%	1.95	40.69	2.38	3.65
**2006**	40,604	2.0%	5.0%	13.5%	2.16	43.23	2.75	4.54
**2007**	42,230	2.1%	5.1%	13.4%	2.37	46.53	3.23	4.67
**2008**	45,054	1.9%	4.8%	14.1%	2.15	44.79	3.96	4.79
**2009**	47,809	2.1%	5.0%	14.2%	2.52	50.32	4.60	5.00
**2010**	51,081	2.1%	4.9%	13.7%	2.37	48.31	**-**	5.29

S-DDD: Defined daily doses for statistical purposes per million inhabitants per day.

NMPOU: Non-medical prescription opioid use.

PO: Prescription opioid.

### Analytic strategy

The primary analytic strategy involved the computation of Pearson’s product moment correlations. As such, r-values and their respective 95% confidence intervals were estimated for the correlations between consumption level of POs and each of the PO-related harms detailed previously, viz.: NMPOU prevalence (past month, past year and lifetime), average number of days per year per person of NMPOU (among the general population and NMPOU users), substance abuse treatment admissions for POs per 10,000 people and PO overdose deaths per 100,000 people.

## Findings

**Figure 1:**
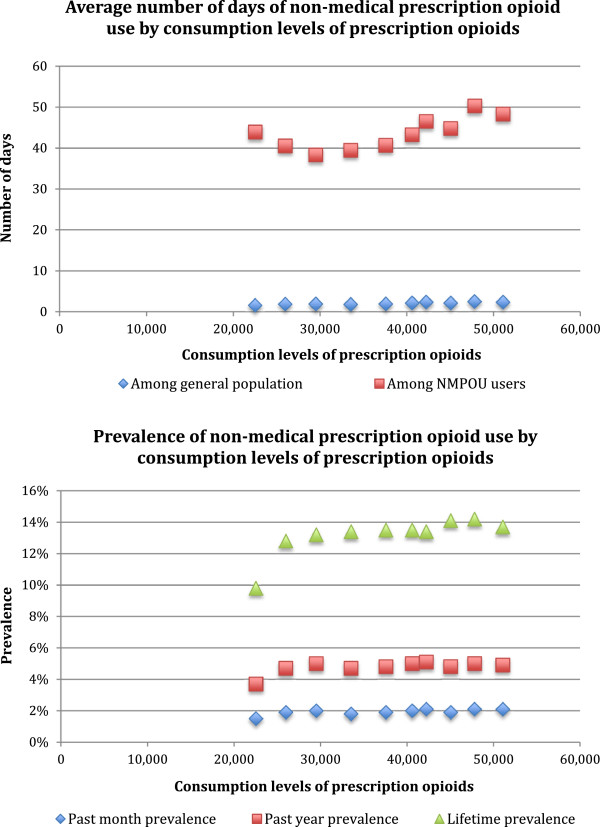
**Visualization of relationships between consumption levels of prescription opioids and non-medical prescription opioid use measures.**

**Figure 2:**
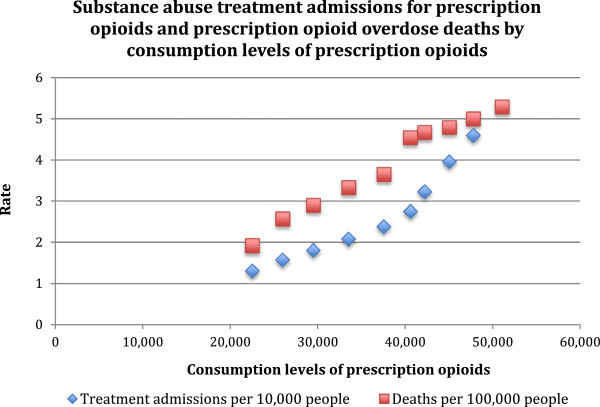
**Visualization of relationships between consumption levels of prescription opioids and prescription opioid-related morbidity and mortality measures.**

Figures 1 and 2 visualize the relationship between consumption levels of POs and each of NMPOU, PO-related morbidity and PO-related mortality measures included in the present study. Table 2 presents the results of the correlational analyses. Based on these data, moderate correlations were observed between consumption levels of POs and prevalence of NMPOU in the past month (r =0.74), past year (r =0.64) or lifetime (r =0.75). However, consumption levels of POs were strongly correlated with average number of days per year per person of NMPOU among the general population and NMPOU users (r =0.90 and 0.72 respectively). Furthermore, consumption levels of POs were very strongly correlated with morbidity and mortality measures, including substance abuse treatment admissions for POs per 10,000 people (r =0.95) and PO overdose deaths per 100,000 people (r =0.99).

**Table 2:** **Correlations between consumption levels of prescription opioids, non-medical prescription opioid use, prescription opioid-related morbidity and prescription opioid-related mortality measures in the United States from 2001 – 2010**

	NMPOU prevalence (past month)	NMPOU prevalence (past year)	NMPOU prevalence (lifetime)	Average number of days per year per person of NMPOU (general population)	Average number of days per year per person of NMPOU (NMPOU users)	Substance abuse treatment admissions for POs per 10,000 people	PO overdose deaths per 100,000 people
**R-value**	0.741	0.638	0.753	0.900	0.720	0.951	0.988
**95% CI**	0.208-0.935	0.014-0.904	0.235-0.938	0.625-0.976	0.165-0.929	0.780-0.990	0.949-0.997

NMPOU: Non-medical prescription opioid use.

PO: Prescription opioid.

## Discussion

This short report documented significant correlations between consumption levels of POs and PO-related harms, including NMPOU, PO-related morbidity and PO-related mortality. These correlations are corroborated by evidence from several other American [8, 9] and Canadian studies [7, 10, 11]. For instance, based on nationally representative data from the United States dating from 1995 to 2004, Wisniewski et al. also documented significant correlations between dispensing levels of two POs and NMPOU prevalence and emergency department visits [9].

However, the magnitude of three correlation coefficients observed in the present study were noteworthy, as near perfect correlations (r >0.90) were observed with consumption levels of POs: average number of days of NMPOU among general population, substance abuse treatment admissions for POs per 10,000 people and PO overdose deaths per 100,000 people. The strength of these correlations indicate linear increases in PO-related harms with increases in consumption levels of POs. Such near perfect correlations have been demonstrated previously for PO-related morbidity in the United States [6], but to our knowledge not for NMPOU or PO-related mortality. Importantly, these findings suggest that in comparison to prevalence estimates of NMPOU, average number of days of NMPOU may be better indicators of NMPOU in the United States. This may partly be due to the stabilization of NMPOU prevalence estimates over the past decade (p >0.05), and the parallel increases (p <0.05) in average number of days of NMPOU over the same time period.

Based on recent data, there seems to be a convergence of evidence implicating increasing consumption levels of POs as the primary system-level driving factor in the surging opioid epidemic. Interestingly, consumption levels of POs have continued to increase throughout the past decade, despite limitations in evidence regarding the effectiveness of pharmacotherapeutic treatment of cancer and chronic non-cancer pain with POs [14, 15]. For example, a review on the effectiveness of long-term opioid management for chronic non-cancer pain concluded that there was only weak evidence to suggest clinically significant reductions in pain [15]. Similarly, the evidence for effectiveness of POs for cancer pain is mixed [14]. Given this current state of the evidence, health policy must weigh benefits of increased consumption levels of POs against the PO-related harms, i.e. potential gains in addressing indications of pain vs. NMPOU, PO-related morbidity and/or PO-related mortality. There exists a need for rigorous reviews of clinical indications that legitimately warrant treatment with POs in comparison to other alternatives. Experts must debate whether there is a genuine need to treat as many individuals as currently treated with POs in the United States.

Irrespectively, the link between consumption levels and public health relevant harms of POs is similar to that observed with other psychoactive substances, including alcohol and tobacco [16, 17]. This link persists in the opposite direction as well when the availability of POs is decreased, i.e. reductions in availability coincide with reductions in PO-related harms [18–20]. These findings emphasize that increased availability of psychoactive substances, including medications, should always be considered in a broader context, encompassing both a clinical and public health perspective.

A key limitation of the present study pertains to the inability to infer causation from the correlations presented. For example, closer surveillance due to increasing public health interest may be responsible for the observed increases in consumption levels of POs or PO-related harms, which would inevitably result in positive correlations. In summary, using data from the United States dating from 2001 – 2010, correlations between consumption levels of POs and PO-related harms were documented. These findings suggest that curbing consumption levels of POs at the population level may be an effective strategy towards limiting PO-related harms.

## Abbreviations

PO: Prescription opioid
NMPOU: Non-medical prescription opioid use
S-DDD: Defined daily doses for statistical purposes per million inhabitants per day
INCB: International Narcotics Control Board.
